# Pragmatic Criteria for Early Discharge After Laparoscopic Colorectal Surgery: Safety and Applicability Outside ERAS Programs

**DOI:** 10.3390/jcm15135205

**Published:** 2026-07-03

**Authors:** Daniele Sandonà, Nicola Passuello, Ugo Grossi, Andrea Grego, Fabrizio Vittadello, Alvise Frasson, Andrea Caudera, Enzo Mammano, Giacomo Sarzo

**Affiliations:** 1 Department of Surgical Oncological and Gastroenterological Sciences—DiSCOG, University of Padua, 35131 Padua, Italy; daniele.sandona.2@studenti.unipd.it; 2 OSA General Surgery, Padua University Hospital, University of Padova, 35127 Padua, Italy; nicola.passuello@aopd.veneto.it (N.P.); fabrizio.vittadello@aopd.veneto.it (F.V.);; 3 Proctology and Pelviperineology Unit, Humanitas Gavazzeni, 24125 Bergamo, Italy; 4 Department of Medicine—DIMED, University of Padova, 35128 Padua, Italy

**Keywords:** early discharge, ERAS, colorectal surgery, complications, minimally invasive

## Abstract

**Background/Objectives**: Enhanced Recovery After Surgery (ERAS) protocols improved outcomes in colorectal surgery, but global implementation remains heterogeneous. In centers without structured ERAS programs, the lack of standardized discharge criteria could lead to conservative decisions and prolonged hospital stays. This study aimed to evaluate the feasibility and safety of early discharge (ED) on postoperative day (POD) 3 using the five Tavernier’s criteria in a real-world setting without formal ERAS pathways. **Methods**: This retrospective analysis of a prospectively maintained database included all consecutive adult patients undergoing elective laparoscopic colorectal resection between February 2025 and February 2026 at a high-volume tertiary center. Patients were stratified into the EARLY group (discharged on POD 3 upon fulfilling all five Tavernier criteria: C-reactive protein < 150 mg/L, temperature < 38 °C, passage of flatus, Visual Analogue Scale score < 5, and oral diet tolerance) and the STANDARD group (discharged after POD 3). The primary endpoint was the safety and negative predictive value (NPV) of the five-criteria bundle regarding 30-day complications. **Results**: Seventy-seven patients were included (EARLY: *n* = 44; STANDARD: *n* = 33). In the STANDARD group, the primary barriers to discharge were prolonged intravenous analgesic requirements (81.8%) and delayed bowel function (36.4%). The five-criteria bundle demonstrated an NPV of 84.1%, a sensitivity of 68.2%, and a specificity of 67.3% for identifying patients at low risk of complications. The overall 30-day complication rate was significantly lower in the EARLY group compared to the STANDARD group (15.9% vs. 45.5%; *p* = 0.010). No major complications (Clavien–Dindo ≥ III) occurred in the EARLY group compared to 6.1% in the STANDARD group. **Conclusions**: This exploratory feasibility analysis suggests that early discharge on POD 3 guided by the five Tavernier criteria is potentially safe and feasible in a real-world clinical setting without formal ERAS pathways. However, given the small sample size and inherent methodological biases, these findings remain preliminary, and larger prospective multi-center trials are strictly required to validate the safety and formal impact of this strategy.

## 1. Introduction

Globally, colorectal cancer represents the third most frequent malignancy, and surgery serves as a critical curative modality [1]. The scope of colorectal surgery also encompasses various benign gastrointestinal pathologies, such as diverticular disease, Crohn’s disease and ulcerative colitis [2]. Nevertheless, universal access to high-quality surgical care is hindered by geographic and economic disparities, leading to unequal clinical outcomes and a substantial socio-economic burden on both healthcare systems and patients [3].

Enhanced Recovery After Surgery (ERAS) programmes have significantly improved perioperative management and accelerated postoperative recovery after colorectal surgery [4]. Core ERAS components include preoperative patient counselling, avoidance of prolonged fasting, opioid-sparing analgesia, early oral intake, early mobilization, standardized fluid management, and predefined discharge planning [5]. When successfully implemented, these pathways have been associated with shorter length of hospital stay, faster functional recovery, fewer complications, and reduced healthcare costs after colorectal resection [6,7].

Regrettably, the global implementation of ERAS protocols remains highly inconsistent due to various systemic barriers [8]. In Africa, the adoption of these programs is still noticeably limited [9]. Similarly, across China, ERAS implementation is fragmented and heterogeneous [10,11]. Furthermore, this implementation gap is not exclusive to developing regions; advanced healthcare systems like those in South Korea and France also exhibit limited and heterogeneous compliance, demonstrating that ERAS principles have yet to be universally translated into real-world clinical practice [12,13].

Several studies have confirmed the safety of early discharge, potentially as early as the third postoperative day (POD 3), and in selected cases even within 24 h [14,15,16]. Moreover, decreasing the LOS has been shown to optimize hospital resource utilization, translating into significant institutional cost reductions [17]. Full adherence to ERAS protocols requires multidisciplinary coordination, dedicated nursing and anaesthesiology engagement, patient education, continuous audit, and institutional commitment. These requirements may represent relevant barriers, particularly in centres where structured ERAS teams, prehabilitation programmes, or post-discharge monitoring systems are not fully available [18,19]. In such contexts, clinicians frequently rely on subjective judgment rather than standardized discharge criteria, this heterogeneity in practice may result in unnecessarily prolonged hospital stays [20]. This lack of validated discharge criteria often leads to conservative clinical decisions, as surgeons may hesitate to authorize early discharge due to concerns regarding potential postoperative complications. A recent consensus report has attempted to mitigate this issue, aiming to standardize methodologies and mitigate heterogeneity among clinical studies [21]. To safely discharge a patient, the physician must be confident that the probability of developing major complications after discharge is extremely low.

C-reactive protein (CRP) levels measured on POD 3 have been shown to possess a high negative predictive value (NPV) for postoperative adverse events following colorectal surgery [22,23]. Nevertheless, CRP alone cannot fully capture the multidimensional nature of postoperative recovery. Safe discharge also requires clinical stability, adequate pain control, return of bowel function, tolerance of oral intake, and absence of systemic signs of infection.

In this context, Tavernier et al. [24] identified and validated a set of five clinical and biochemical criteria to guide safe discharge after colorectal surgery. However, these criteria were originally evaluated within strict ERAS pathways, and their applicability in centers without structured ERAS protocols remains uncertain.

The aim of this study was therefore to evaluate the feasibility and safety of early discharge within 3 days in a real-world clinical setting where formal ERAS programmes are not systematically implemented, applying the 5 criteria proposed by Tavernier: a CRP level below 150 mg/L, absence of fever during the entire hospital stay (temperature < 38 °C), resumption of bowel function (passing gas), adequate pain control with oral analgesics (Visual Analogue Scale (VAS) score < 5), and tolerance of an oral diet. The primary objective was to assess whether this simple clinical bundle could safely identify patients at low risk of postoperative complications within 30 days after surgery.

## 2. Materials and Methods

### 2.1. Study Design and Population

This study is a retrospective analysis of a prospectively maintained database, conducted at the General Surgery Unit of Padua University Hospital. The study was conducted in accordance with the Declaration of Helsinki, and approved by the Ethics Committee Area Centro-EST Veneto (CET code 6586n/AO/26 on 19 March 2026). Informed consent was obtained for each patient. We included all consecutive adult patients who underwent elective laparoscopic colorectal resection between February 2025 and February 2026. Patients undergoing emergency surgery, open procedures, or conversion to laparotomy were excluded. No patients were excluded as there were no missing data. The study reflects routine clinical practice in a high-volume tertiary referral centre where early discharge (ED) is considered for patients demonstrating optimal postoperative recovery. Patients were stratified into two groups based on the length of hospital stay (LOS): the EARLY group, comprising patients discharged on POD 3, and the STANDARD group, including patients discharged after POD 3.

### 2.2. Early Discharge Criteria

In our institution, eligibility for ED following laparoscopic colorectal surgery is assessed on POD 3 using the Tavernier’s criteria. Patients fulfilling all five criteria are routinely discharged on this day, in accordance with institutional practice.

### 2.3. Perioperative Management

All patients received traditional post-operative care, rather than a specific ERAS program. Preoperative mechanical bowel preparation was administered only to patients undergoing left-sided resections, rectal resections, or Hartmann’s reversal. Aside from this aspect, the perioperative management was identical for both colon and rectal resections. A prophylactic abdominal drain was routinely placed in all patients, and reinforcement sutures were performed whenever a colorectal anastomosis was created. Although a formal ERAS pathway was not implemented, certain standard perioperative practices aligned with ERAS principles were routinely applied, such as early mobilization, early removal of the urinary catheter, and omission of routine nasogastric tubes. Nevertheless, these were not part of a standardized protocol, and their specific timing and modalities were left entirely to the operating surgeon’s discretion. Oral fluid intake was initiated immediately after the patients returned to the ward, with diet advancement based on individual tolerance. Intravenous fluid administration was routinely discontinued on POD 1, provided no signs of ileus were present. Biochemical assessments, including CRP levels, were routinely performed on POD 1 and POD 3, with additional laboratory tests performed at the surgeon’s discretion. Pain management consisted of intravenous analgesia until POD 1, followed by a transition to oral agents, tailored to individual tolerance. Opioid use was minimized to facilitate bowel recovery but not entirely excluded. Routine imaging was not required for discharge unless clinically indicated by a deviation from the expected recovery path.

### 2.4. Data Collection and Outcomes

Clinical data were retrospectively extracted from a prospectively maintained electronic database. The collected variables included baseline characteristics such as age, sex, body mass index (BMI), smoking status, ASA physical status, and the Charlson Comorbidity Index (CCI). Intraoperative data focused on the procedure type and operative time, while postoperative metrics included timing of adequate pain management with oral analgesic, timing of bowel function recovery, diet tolerance, and CRP levels on POD 1 and POD 3.

The primary endpoint was the safety of discharge, evaluated through the diagnostic performance and negative predictive value (NPV) of the five-criteria bundle in identifying patients at low risk for postoperative complications. Secondary endpoints included the comparison of 30-day complication rates and readmission rates between groups. The success of the early discharge strategy was strictly defined as discharge on POD 3 without the development of complications or the need for readmission within 30 days. All complications were graded according to the Clavien–Dindo classification and monitored for up to 30 days postoperatively.

### 2.5. Statistical Analysis

Continuous variables are presented as median and interquartile range (IQR), while categorical variables are reported as absolute frequencies and percentages. The diagnostic accuracy of the five discharge criteria was assessed by calculating sensitivity, specificity, positive predictive value (PPV), and NPV. To handle the small sample size, Firth’s penalized logistic regression was employed to identify preoperative factors associated with the development of postoperative complications. To reduce selection bias between groups, an Inverse Probability of Treatment Weighting (IPTW) approach was attempted. Given the exploratory nature of the study, no formal sample size calculation was performed. Statistical analyses were performed using the Python programming language, version 3.12 (Python Software Foundation, https://www.python.org (accessed on 31 May 2026)) [25]. Statistical significance was set at *p* < 0.05.

## 3. Results

### 3.1. Patients Characteristics

The study population consisted of 77 patients, divided into the EARLY group (*n* = 44) and the STANDARD group (*n* = 33). The two groups were comparable regarding age [median 64.5 (IQR 57.5–74) vs. 71 (60–81) years; *p* = 0.073] and comorbidity burden, with a median CCI of 3 in both cohorts [EARLY: 3 (IQR 2–3); STANDARD: 3 (IQR 2–5)]. Conversely, statistically significant differences were observed in terms of BMI [24.5 (IQR 22.7–26.4) vs. 23 (IQR 21–25) kg/m^2^; *p* = 0.016] and ASA score [median 2 (IQR 2–2) vs. 2 (IQR 2–3); *p* < 0.001] (Table 1). However a clinically significant effect size was observed exclusively for the ASA score that shows an r = 0.383 (0.121–0.645). The most common surgical procedure performed was left hemicolectomy (*n* = 33, 43%), followed by right hemicolectomy (*n* = 24, 31%), rectal resection (*n* = 13, 17%), subtotal colectomy (*n* = 5, 6%) and Hartmann reversal (*n* = 2, 3%). Indications included 44 neoplasms (57%) and 33 benign conditions (43%).

### 3.2. Postoperative Recovery and Criteria Achievement

In the EARLY group, 100% of patients met all five discharge criteria, whereas in the STANDARD group, the most common reason for delayed discharge was intravenous analgesic requirement for more than 3 days, observed in 81.8% of cases. Beyond pain control, other significant barriers to discharge in this group included delayed recovery of bowel function (passage of flatus >3 days), observed in 36.4% of cases, and persistent systemic inflammatory response, evidenced by CRP levels ≥150 mg/L in 30.3% of patients. Fever (POD 3) and nutritional issues were less frequent causes of delay, accounting for 12.1% and 9.1% of cases respectively.

Multivariable Firth’s penalized logistic regression analysis was performed to identify preoperative factors associated with the development of postoperative complications (Table 2). None of the variables were statistically significant (*p* < 0.05), though a borderline trend was observed for the ASA score (*p* = 0.065). However, due to the limited sample size, IPTW did not achieve a fully satisfactory balance of baseline characteristics across the groups.

### 3.3. Primary Outcome: Safety and Predictive Performance

The five-criteria bundle demonstrated a NPV of 84.1% for identifying patients who could be safely discharged without 30-day complications. The sensitivity and specificity were 68.2% and 67.3%, respectively (Table 3).

### 3.4. 30-Day Clinical Outcomes

The overall 30-day complication rate was 28% (*n* = 22). In the EARLY group, 7 patients (15.9%) experienced a complication within 30 days, compared to 15 patients (45.5%) in the STANDARD group (*p* = 0.010). Major complications (i.e., Clavien–Dindo ≥ III) occurred in 0 patients in the EARLY group and 2 (6.1%) in the STANDARD group. The readmission rate within 30 days was 2.3% (*n* = 1) in the EARLY group and 6.1% (*n* = 2) in the STANDARD group. The mortality rate within the 30-day follow-up period was 0% (*n* = 0) in the EARLY group and 3% (*n* = 1) in the STANDARD group. Successful early discharge was achieved in 84.1% of the EARLY group.

Beyond overall incidence, the severity profile of complications differed between the two groups. In the EARLY group, all recorded complications (*n* = 7) were self-limiting Clavien–Dindo I–II, including bowel obstruction, tenesmus, surgical site infection, urinary tract infection, fever during outpatient follow-up, constipation, and positioning-related hand hypoesthesia.

In contrast, the STANDARD group presented with a broader and more severe spectrum of complications, including four wound infections, four cases of fever, three bowel obstructions, one intestinal perforation, one pleural effusion, one bleeding event requiring blood transfusion, and one death.

**Figure 1:**
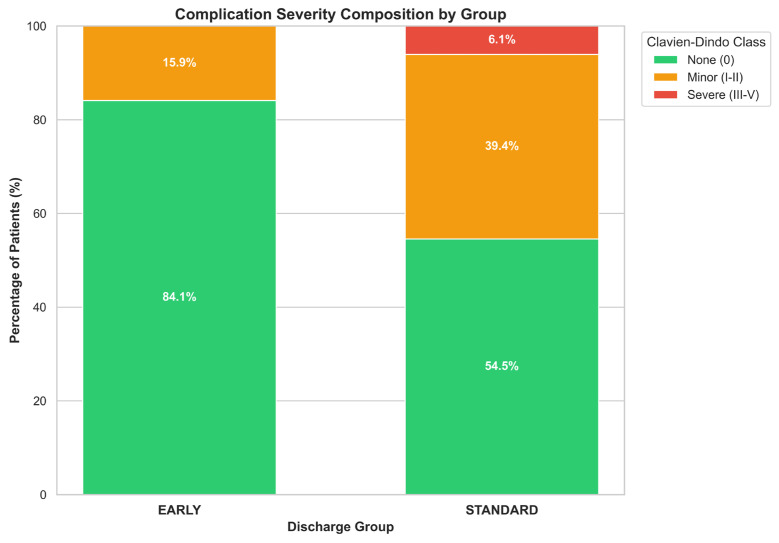
Complication severity composition by group.

The safety analysis confirmed that the rate of missed severe complications in patients meeting discharge criteria was 0.00%. The safety analysis confirmed that the rate of missed severe complications in patients meeting discharge criteria was 0.00% (Figure 1).

## 4. Discussion

The present study demonstrates that a standardized bundle of five clinical and biochemical criteria [24] is a reliable and safe tool for guiding ED after laparoscopic colorectal surgery, even when applied outside the framework of formal ERAS or prehabilitation programs. Our findings are particularly significant because they address a common clinical dilemma: how to identify patients suitable for discharge by POD 3 in centers where complex recovery protocols are not yet fully established. A cornerstone of our strategy is the achievement of objective recovery milestones. In our cohort, the EARLY group (discharged ≤ POD 3) experienced zero major complications (Clavien–Dindo ≥ III) and zero mortality. While 15.9% of these patients did experience a complication within 30 days, it is crucial to note that all were minor (Clavien–Dindo I–II) and manageable in an outpatient setting. While these results translate to a 100% NPV for major complications, the 95% confidence interval is wide (92.0–100.0%) due to our small sample size. This statistical uncertainty is clinically meaningful: the absence of events in a sample of 44 patients cannot definitively rule out the risk of severe post-discharge complications in a larger, unselected population and must be interpreted with caution.

The choice of a single CRP threshold (<150 mg/L) on POD 3 proved to be a pragmatic and effective ‘rule-out’ tool. While some literature advocates for evaluating CRP trajectories or more sensitive markers like procalcitonin [26], a single, clear cut-off was highly discriminative in our series. Interestingly, the STANDARD group showed significantly higher CRP levels on POD 3 (median 88 vs. 58.5 mg/L), suggesting that persistent systemic inflammation is a reliable proxy for subclinical recovery delays. Analysis of the STANDARD group revealed that the primary barrier to discharge was the persistent requirement for intravenous analgesia (81.8%). This highlights that, in the absence of a formal ERAS pathway, postoperative pain management remains the ‘weakest link’ that prevents earlier discharge. However, the fact that the EARLY group achieved discharge safely despite this indicates that a subset of patients, even those with a higher BMI as seen in our EARLY cohort, can recover rapidly through standard minimally invasive techniques and basic accelerated care.

An important aspect of our findings is their applicability to everyday clinical practice. Most studies evaluating early discharge after colorectal surgery have been conducted in institutions with mature ERAS programmes, dedicated multidisciplinary teams, and highly standardized perioperative pathways [14,15,16,24]. While these represent the ideal environment for accelerated recovery, they may not accurately reflect the reality of many surgical units worldwide. In contrast, our study was intentionally conducted in a setting where there was no formal ERAS implementation. Nevertheless, a substantial proportion of patients were safely discharged by POD 3, suggesting that complete ERAS adoption may not be an absolute prerequisite for achieving short hospital stays after minimally invasive colorectal surgery. Rather, the identification of objective recovery milestones may represent the critical factor. This observation is potentially relevant for healthcare systems facing increasing pressure on hospital bed capacity and resource allocation. However, because our study did not directly collect institutional cost or bed-day data, claims regarding formal resource optimization remain purely speculative. A simple bedside discharge checklist may therefore provide a practical and immediately applicable strategy for centers wishing to optimize postoperative care while gradually progressing toward full ERAS implementation.

The five criteria included in the bundle directly reflect the fundamental domains of postoperative recovery, i.e., systemic inflammation, gastrointestinal function, nutritional tolerance, pain control, and clinical stability. Consequently, they offer an intuitive and transparent framework that can be readily understood and applied by surgeons, nursing staff, and trainees without the need for specialized software or advanced statistical expertise.

Finally, the finding that no patient meeting all five criteria developed a major complication deserves particular attention. Although the overall NPV for any complication was 84.1%, the absence of Clavien–Dindo grade III or higher complications among eligible patients suggests that the bundle may be especially useful as a safety tool rather than merely a predictor of postoperative events. From a clinician’s perspective, the primary concern when considering discharge is not the occurrence of minor self-limiting complications but the risk of missing serious adverse events requiring invasive treatment or urgent readmission. Therefore, the observed 100% [92–100%] NPV for major complications offers a preliminary indication of safety, supporting the further evaluation of this bundle as a pragmatic and conservative rule-out strategy for severe postoperative morbidity.

### Limitations

Our study has several fundamental methodological limitations that restrict the generalizability of the findings. First, it is an exploratory feasibility analysis with a very small sample size (*n* = 77), which severely limits statistical power. Second, the study design suffers from a critical circularity bias: because the five Tavernier criteria were already embedded in our institutional routine to guide discharge decisions on POD 3, patients were selected for early discharge precisely because they met these criteria, automatically confounding the correlation between criteria achievement and the timing of discharge. Third, significant selection and confounding biases are present; the EARLY and STANDARD groups exhibited baseline imbalances, particularly regarding the ASA score, and due to the small cohort size, our Inverse Probability of Treatment Weighting (IPTW) approach failed to achieve a satisfactory balance between groups.

## 5. Conclusions

In conclusion, this exploratory feasibility analysis suggests that early discharge by POD 3 is potentially safe and feasible following laparoscopic colorectal surgery, provided that a specific bundle of five clinical and biochemical criteria is met. This strategy shows promise in identifying patients at low risk for major complications, even without the systematic implementation of formal ERAS protocols. However, due to severe limitations—including a small sample size, significant circularity and confounding biases, and the lack of direct cost or bed-day data—our findings cannot be considered definitive evidence of proven safety or resource optimization. Larger, prospective, multi-center studies with rigorous bias adjustment are strictly warranted to validate these criteria and formally evaluate their clinical and economic impact.

## Figures and Tables

**Table 1:** This table presents the main characteristics of the two groups.

Variable	EARLY	STANDARD	*p* Value
Age (years)	64.5 (57.5–74)	71 (60–81)	0.073
BMI (kg/m^2^)	24.5 (22.7–26.4)	23 (21–25)	0.016
CCI	3 (1.8–3)	3 (2–5)	0.106
ASA	2 (2–2)	2 (2–3)	<0.001
Operation duration (minutes)	188.5 (153.8–231.2)	220 (165–255)	0.158

**Table 2:** Firth’s penalized logistic regression analysis of factors associated with the presence of post-discharge complications.

Variable	Odds Ratio	95% CI	*p* Value
Age	1.001	[0.958–1.046]	0.956
Sex F	1.433	[0.503–4.082]	0.501
Rectal resection	1.747	[0.591–5.165]	0.313
ASA	2.235	[0.838–5.958]	0.108

**Table 3:** Diagnostic accuracy of five clinical criteria. FNR (false negative ratio), NPV (negative predictive value), PPV (positive predictive value).

Metrics	Eligibility
Sensitivity	68.2% [47.3–83.6%]
Specificity	67.3% [54.1–78.2%]
FNR	31.8% [16.4–52.7%]
NPV	84.1% [70.6–92.1%]
PPV	45.5% [29.8–62.0%]

## Data Availability

The data presented in this study are available on request from the corresponding author due to privacy concerns.
